# Disruptive Behavior Programs on Primary School Students: A Systematic Review

**DOI:** 10.3390/ejihpe10040070

**Published:** 2020-10-16

**Authors:** Diego Martín Retuerto, Iker Ros Martínez de Lahidalga, Irantzu Ibañez Lasurtegui

**Affiliations:** Faculty of Education and Sports, University of the Basque Country (EHU/UPV), 01006 Vitoria-Gasteiz, Spain; diego.martin_10@hotmail.com (D.M.R.); irantzu.ibanez@ehu.eus (I.I.L.)

**Keywords:** disruptive behavior, childhood, pre-adolescence, emotional management

## Abstract

The objective of this study was to review the existing international literature on research and programs for the reduction of disruptive behavior in primary school students. For this purpose, according to PRISMA-ScR, a mixed systematic review was performed in six databases in order to obtain wide and extensive information related to the subject under study. The studies obtained were analyzed through a table which emphasized the data related to: Author(s), year, educational stage, location, objectives, instruments, and results. As for the selection of studies, the UNESCO Thesaurus and the ERIC Thesaurus terminology was used. In addition to specifying the search for studies performed between 2004 and 2020 (both inclusive), articles written in Spanish and English were selected. Furthermore, in a final phase among the articles analyzed, those that were not or did not contain intervention programs were discarded. Therefore, a total of thirty-five articles out of more than twenty thousand were analyzed in depth. The results showed that a majority of programs were implemented in the primary education stage, as well as a predominance of the use of instruments, such as questionnaires and observation charts. In addition, it is important to underline that 77.14% of the programs analyzed were effective, hence, they met the proposed objectives. In summary, although the number of intervention programs for the reduction of disruptive behavior that can be found in the international scientific literature is growing, there is still a long way to go in order to create a large network that can serve as a foundation for interventions in primary education students.

## 1. Introduction

Today, we find ourselves at a time when technological development and globalization are advancing so rapidly that we humans are often unable to adapt [1]. In the same way, there are new challenges and demands that require other modes of school organization, new responsibilities and roles for teachers, as well as the acquisition of skills that allow them to adapt to continuous change [2]. Taking that into account, different authors [1,3,4] indicate that there should be a good climate of coexistence to favor healthy interpersonal relationships among the members involved in the educational community (teachers, families and students), “for which we recommend citizen training, working on dialogue as a learning tool and family-school co-responsibility” [5].

Linked to these interpersonal relationships are the behaviors that are developed in the educational environment. It should be noted that in Primary Education, the predominant behaviors that cause a distortion in the order are noise and verbal and aggressive behavior, being in the last years of this educational stage where these take greater relevance [6]. In the same line, several authors [7,8] recommend that programs to prevent and correct disruptive behaviors should be implemented in the first years of life to avoid greater problems in the future. Interestingly, other authors [9] suggest that parental education style may be one of the sources generating such behaviors.

In relation to the subject of study, in previous research performed by Ros [10,11] and Rodriguez et al. [12], it was found that “engagement”, and specifically its behavioral dimension, is closely related to disruptive behaviors. In detail, a lack of involvement could lead to serious behavioral problems and an increase in disruptive behaviors.

With regard to bullying in particular, it should be noted that, as indicated by Halabi et al. [13], even though mental illness can be an aggravating factor in its occurrence, the main factors in the explanation of bullying, following the socio-ecological model, are school, family, and individual levels. In relation to diseases, Mendez, et al. [14] suggested that working on emotional intelligence could be a useful and effective tool and should be integrated into programs against bullying.

In relation to teacher training, Álvarez Martino et al. [15] observed that 47.4% of teachers in Spain did not have specific training in disruptive behavior programs, which in other countries are almost a requirement. Therefore, institutional support for teacher programs is needed since such training would lead to an improvement in the quality of the educational system.

In the following review, disruptive behavior is analyzed from a more general perspective and not exclusively focused on bullying. In other words, we understand disruptive behavior as a set of behaviors that generate adaptations in school sessions and require interventions by external and/or internal agents. These behaviors could range from talking or teasing to even physical or psychological aggression.

Nevertheless, as bullying is one of the consequences of disruptive behavior, further research should be needed to tackle this issue. In addition, it is necessary to examine the different conflicts in the school institution and their main causes which could be ideological-scientific, power-related, structure-related and/or related to personal and interpersonal relationship issues [16]. At the same time, it is important to note that due to the broad terminology, specific terminology obtained through the UNESCO Thesaurus and ERIC Thesaurus encyclopedias was used in this search, such as “social maladjustment program,” “disruptive behavior program,” and “antisocial behavior” among others (see Table 1). Thus, despite the shortage of literature in this subject [17], the intention is to disseminate different strategies and/or programs that can be used to manage disruptive behavior in the school setting. 

Moreover, an attempt to collect varied experiences has been made in order to provide a potential transference to the three main learning contexts (formal, non-formal and informal education) [18] and the different educational stages (from Infant Education to University).

In summary, the main objective of this study is to shed light on international knowledge by conducting a literature review on programs and research for the reduction of disruptive behavior, focusing specifically on different learning contexts. With regard to the specific objectives, geographic origin, evolution of the number of studies over time, educational stage, instruments used, and the results of the implemented programs in terms of the degree of fulfillment of the objectives will be examined.

## 2. Materials and Methods 

The review was conducted according to PRISMA-ScR (Preferred Reporting Items for Systematic Reviews and Meta-Analyses) [19] following a protocol [17] and a structure [20] similar to others. In order to reduce selection bias, two independent reviewers performed title, abstract and full article screening in parallel. Moreover, data extraction from the included studies was conducted by two independent reviewers.

### 2.1. Search Limits

A mixed systematic search [21] of six databases (Google Scholar, Dialnet, Scopus, Pubmed, Web of Science and ERIC Thesaurus) was performed (2004–2020) in order obtain extensive and comprehensive information on the subject under study. These databases were selected as they included articles published in journals indexed in the Journal Citation Report (JCR) or the Scimago Journal Rank (SJR). 

### 2.2. Selection Criteria

The criteria for including studies were as follows: (a) Studies containing the words “program” and “disruptive behavior” in both English and Spanish or, “Social Inadaptation Program” and “Disruptive Behavior Program”, (b) studies published from 2004 to 2020 (both inclusive), (c) studies that included quantitative and/or qualitative methods and findings, (d) research conducted in school settings, (e) in the ERIC Thesaurus, studies published from 2015 to 2020 (both inclusive) and that the full text was available.

In the identification process, a selection was made with the keywords (see Table 1) and the search was limited to documents in both Spanish and English containing the words “program” and “disruptive behavior”.

In the first selection, words related to the subject were searched through the UNESCO Thesaurus, which yielded the following results:

i. “Conducta antisocial” (Anti-Social behavior), “Inadaptación” (Inadaptation), “Trastornos de la personalidad” (Personality disorders), “Adaptación social” (Adaptación social), “Alienación social” (Social alienation), “Delincuencia juvenil” (Juvenile delinquency), “Comportamiento antisocial” (“Anti-social behavior“), “Conducta antisocial” (Anti-social conduct), “Inadaptación social” (Social Inadaptation)
ii. “Disrupción” (Disruption): No results were found.
iii. “Programa” (Program): Several types were found and “Programa de Educación” (Educational Program) was selected.

These results were applied in databases that contained documents written in Spanish (Google Scholar and Dialnet) in order to perform the first screening. 

A second search (screening 2) used the ERIC Thesaurus, Google Scholar, Web of Science, Scopus and PubMed using the terms “Social Maladjustment Program” and “Disruptive Behavior Program” all obtained from the ERIC Thesaurus. It is important to note that in the ERIC Thesaurus, the search was limited to the last five years and to articles with the full text available.

After the identification and the first selection, more than 517 articles were selected as potential studies. After the second selection, 37 documents were obtained, which matched the terminology and met the requirement of being an intervention program. Finally, after a complete reading (inclusion stage), two of them were discarded, in one of the cases for not containing or being a program and, in the other case, for being just a protocol. Therefore, the sample finally analyzed was of 35 articles.

### 2.3. Data Extraction

Articles that did not fit the publication date were discarded in the first level. Articles that met the selection criteria were retrieved for this review. Articles were classified as follows: “Author(s)”, “year”, “educational stage”, “location”, “objectives”, “instrument(s)”, and “results”. A table (see Table 2) was used to collect the documents and filter the results.

### 2.4. Data Analysis

**Figure 1:**
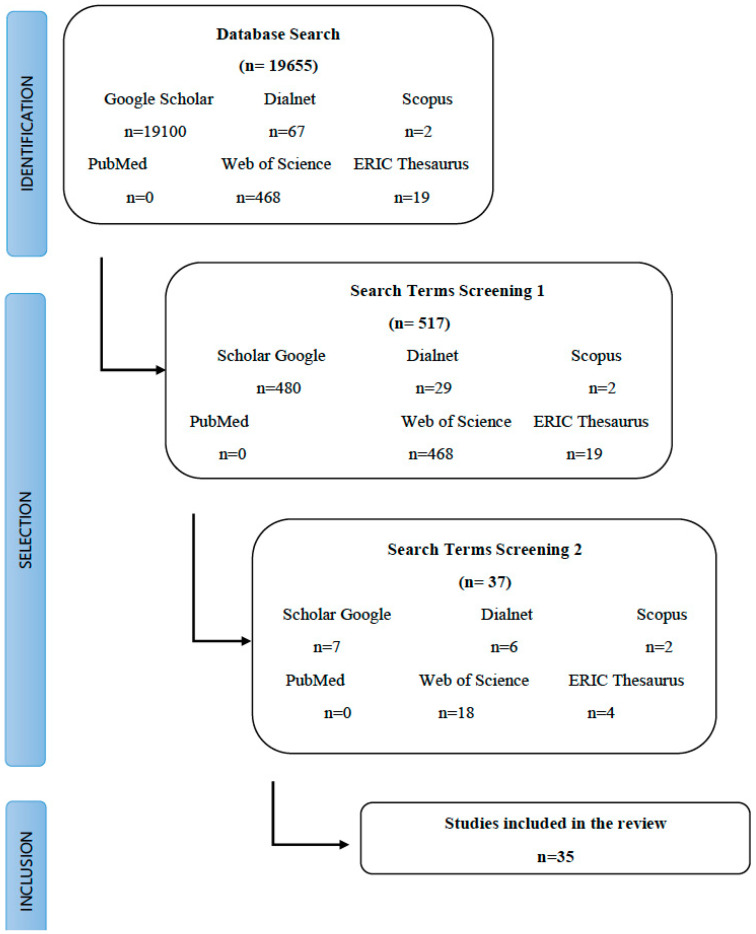
Search and inclusion process for a scoping review on disruptive behavior.

Considering the terminology and the steps followed, already described in point 2.2, the summary of the process can be seen in Figure 1. In summary, the steps followed to achieve the results were as follows:

**i.**  Identification: Selection of databases (Google Scholar, Dialnet, Scopus, PubMed, Web of Science and ERIC Thesaurus) and general terminology (“conducta disruptiva” and “disruptive behavior”).
**ii.**  Screening 1: Selection of terminology in Spanish (see Table 1) and specific search in databases with Spanish documents (Google Scholar and Dialnet).
**iii.**  Screening 2: Selection of terminology in English (see Table 1) and specific search in the databases with documents in English (Google Scholar, Scopus, Pubmed, Web of Science and ERIC Thesaurus).
**iv.**  Inclusion: Reading and complete analysis of the documents and final selection where the fundamental requirement was to contain an intervention program.

## 3. Results

The information was structured in a similar way to that used by Montoya Fernández [17]. Figure 1 shows the flow chart followed during the search process.

Out of more than 20,000 results, a total of 35 were analyzed in-depth (see Table 2).

### 3.1. Study Locations and Evolution Over Time

The place of application of the programs included studies from Spain (13), followed by the United States (5), then a group of countries where the Finnish anti-bullying program ‘KiVa’ was applied: Finland (4), United Kingdom (3), Italy (3), and the Netherlands (3), and finally Chile (2), Austria (1), Australia (1), Bolivia (1), Canada (1), Colombia (1), Ecuador (1), Germany (1), Mexico (1), Peru (1), and some others (5) which have an “undetermined” location.

**Figure 2:**
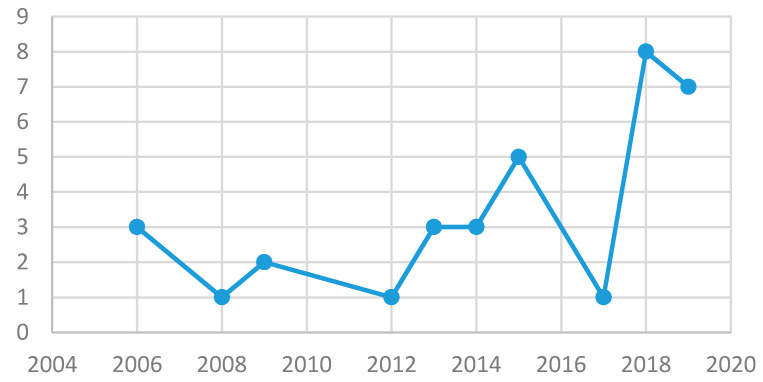
Evolution of number of studies over time.

Regarding the evolution of the studies by year of publication (Figure 2), it can be observed how the number of publications has been progressively increasing throughout the years, but without yet reaching the barrier of the ten annual prevention programs.

### 3.2. Educational Stage of the Participants

**Figure 3:**
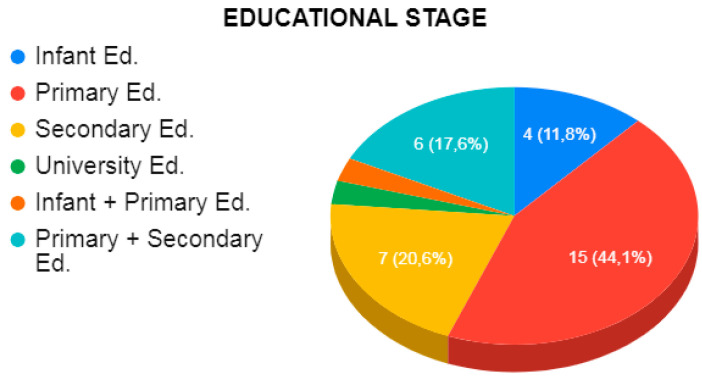
Educational stage of the participants.

Considering the educational stage in which the programs were applied (Figure 3), of the thirty-five studies, four exclusively were for infant education (11.42%), fifteen exclusively for primary education (42.85%), seven exclusively for secondary education (20%), one exclusively for university education (2.85%), one for infant and primary education (2.85%), and six for primary and secondary education (17.14%).

### 3.3. Instruments

**Figure 4:**
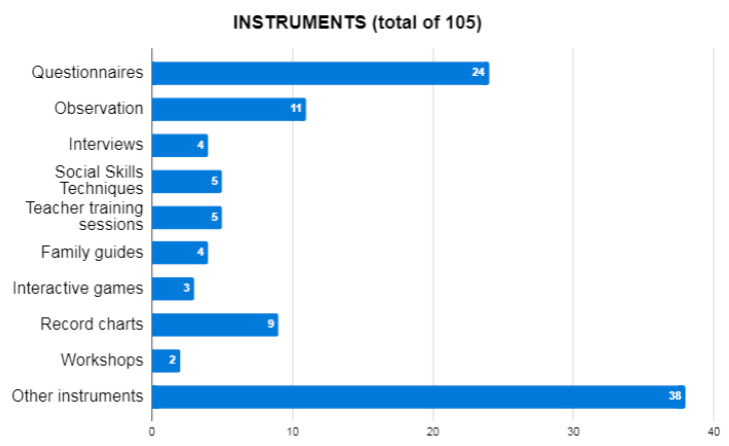
Instruments used in the reviewed studies.

A total of 105 records were obtained with regard to the instruments used in the studies (Figure 4). The most common instruments were questionnaires (22.86%), observation (10.48%), record charts (8.57%), interviews (3.81%), social skills techniques (4.76%), teacher training sessions (4.76%), family guides (3.81%), interactive games (2.86%), workshops (1.9%), and other more specific instruments (36.19%), such as albums, mobile phones, “the color wheel”, videos and cartoons, games, peer work, or education consultants. 

### 3.4. Outcome Measures

**Figure 5:**
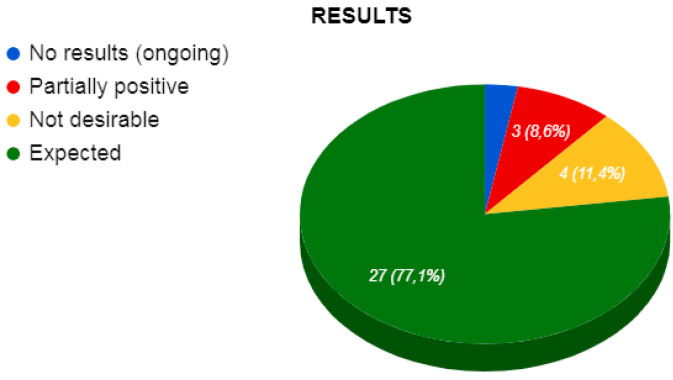
Outcome measures of the studies reviewed.

Regarding the results of the studies analyzed (Figure 5), in one of the studies, neither positive nor negative results could be obtained because it was in the process of implementation, in three of them, results were partially positive to those expected, in four of them, the desired results were not obtained and in twenty-seven studies, the expected results were obtained. Therefore, it could be said that from a sample of the thirty-five documents analyzed, the complete effectiveness was 77.14%.

## 4. Discussion

The main purpose of this study was to provide knowledge about research and programs implemented for the reduction of disruptive behavior. Based on the results of this research and in line with Montoya Fernandez [17], there is limited relevant literature on disruptive behaviors in the primary education stage. In other words, despite the fact that this is a social problem that is growing considerably, there is no significant literature that can serve as a base for a satisfactory protocol or effective intervention. Nevertheless, most programs were found to be implemented in primary school, which is a good period to work on the prevention of this type of behavior that predominates in the first cycle of the secondary education stage [56,57,58,59].

Relating to the objectives of the studies analyzed, the majority (18 documents) [22,23,25,28,31,32,33,34,35,37,38,40,42,43,46,49,52,55] were intended to reduce disruptive behavior or to improve behavior. However, ten of the studies [5,24,28,29,30,39,41,47,53,54] also attempted to analyze the reasons that led to disruptive behaviors in order to apply the most appropriate techniques. Others (seven studies) simply attempted to evaluate the effectiveness of the implemented program [26,36,44,45,48,50,51].

Regarding the results observed in the effectiveness of the programs, it could be thought that almost any proposal could bring benefits to the behavior of the students. However, it is always advisable to follow guidelines, such as seeking an appropriate classroom atmosphere, motivation, the involvement of families and peers, or the coordination of school staff [6,47,60,61,62,63,64,65].

In terms of the number of disruptive behavior prevention programs according to the educational stage of the participants, it was observed that the largest number of programs are aimed at primary school students, followed by secondary school, nursery school, and university education. Therefore, despite the fact that the vast majority of disruptive behaviors and conflicts occur in the first cycle of secondary education [56,57,58,59], primary education is the ideal stage for their prevention.

In relation to the instruments used, there was a predominance of questionnaires, observation, and record sheets. This was followed by social skills techniques, teacher training, interviews, and family guides, as well as interactive games. Therefore, as in the KiVa program [23], there were three fundamental pillars: Teacher training, guidelines and training for families, and tools for students to improve disruptive behaviors. Therefore, we are once again faced with the triangle that makes up the educational system (student–family–teacher), in which the three pillars are fundamental for sustenance and positive progress.

## 5. Conclusions

Although an increasing number of intervention programs on disruptive behavior can be found in the international literature, there is still a long way to go to create a large network that can serve as a foundation for interventions in primary education students. Therefore, future studies should perform a new revision of the terminology used for the search in order to obtain a greater number of results or more relevant ones. Once the terms have been specified again, both a qualitative and quantitative statistical analysis would be necessary in order to decide more precisely which methods are the most effective.

## Figures and Tables

**Table 1:** Search terms used in each database.

Database	Search Terms
**Used in All Searches**	“program” (“programa”), “disruptive behavior” (“conducta disruptiva”), “social inadaptation program” (“programa inadaptación social”), “disruptive behavior program”, (“programa conducta disruptiva”)
	**Additional Search Terms**
**Google Scholar**	“Anti-Social Behavior“, (“Conducta antisocial”), “Inadaptation“, (“Inadaptación”), “Personality disorders“, (“Trastornos de la personalidad”), “Social adaptation“, (“Adaptación social”), “Social alienation“, (“Alienación social”), “Juvenile delinquency“, (“Delincuencia juvenil”), “Anti-social behavior“, (“Comportamiento antisocial”), “Anti-social conduct“, (“Conducta antisocial”), “Social Inadaptation“, (“Inadaptación social”), “Educational Program“, (“Programa de Educación”)
**Dialnet**	“Anti-Social Behavior“, (“Conducta antisocial”), “Inadaptation“, (“Inadaptación”), “Personality disorders“, (“Trastornos de la personalidad”), “Social adaptation“, (“Adaptación social”), “Social alienation“, (“Alienación social”), “Juvenile delinquency“, (“Delincuencia juvenil”), “Anti-social behavior“, (“Comportamiento antisocial”), “Anti-social conduct“, (“Conducta antisocial”), “Social Inadaptation“, (“Inadaptación social”), “Educational Program“, (“Programa de Educación”)
**Scopus**	“Social maladjustment Program”, “Disruptive Behavior Program”
**PubMed**	“Social maladjustment Program”, “Disruptive Behavior Program”
**Web of Science**	“Social maladjustment Program”, “Disruptive Behavior Program”
**ERIC Thesaurus**	“Social maladjustment Program”, “Disruptive Behavior Program”

**Table 2:** Summary of studies on disruptive behavior.

Author(S) and Year	Educational Stage	Location	Main Objective	Instrument(s)	Results *
University of Turku (2006) [22]	Primary and Secondary Education	Finland, United Kingdom, Italy, Netherlands, Spain	To reduce the number of cases of school bullying through: Teacher training, helping families and educating through values, such as empathy.	- Training sessions for teachers - A guide for families - Interactive games and specific sessions for students - Follow-up questionnaires for students	*●●●*
Martínez R. (2015) [23]	Primary and Secondary Education	Finland, United Kingdom, Italy, Netherlands, Spain	To reduce the number of cases of school bullying through: Teacher training, helping families and educating through values, such as empathy.	- Training sessions for teachers - A guide for families - Interactive games and specific sessions for students - Follow-up questionnaires for students	*●●●*
Pichardo M.C. et al. (2008) [24]	Primary Education	Bolivia	To evaluate the effects of a social skills program, aimed at students in the first, second, and third year of primary education.	- Questionnaires	*●●●*
Coronado A. (2009) [25]	Secondary Education	Undetermined	To improve the behavior of secondary education students who showed disruptive behavior.	- Semi-structured interviews - Observation - Reports and academic records	*●●●*
Corsi E. et al. (2009) [26]	Primary Education	Chile	To evaluate the effectiveness of a behavioral intervention program (“the good behavior game”) in decreasing the frequency of disruptive behavior and increasing the frequency of prosocial behavior in the school setting.	Analysis: - Instructions - Verbal praise - Economy of tokens - Response cost Teacher training: - Role-play - Live modeling - Behavioral Test - Modeling - Self-modeling	*●●●*
Salmivalli C. et al. (2012) [27]	Primary and Secondary Education	Finland, United Kingdom, Italy, Netherlands, Spain	To reduce the number of cases of school bullying through: Teacher training, helping families and educating through values such as empathy.	- Training sessions for teachers - A guide for families - Interactive games and specific sessions for students - Follow-up questionnaires for students	*●●●*
Bierman K. L. et al. (2013) [28]	Primary and Secondary Education	United States	To identify the cognitive and behavioral contributions that lead to disruptive behavior during the school years and to evaluate the impact of the “Fast Track prevention program” in the school setting.	- PATHS (Promoting Alternative Thinking Strategies) - Individual tutoring - Peer-to-peer work - Middle School Transition Program - Academic support in secondary school - After-school training for families and social skills teams for children - Education consultants - Participation questionnaires	*●○○*
Macazaga A. M. et al. (2013) [29]	Primary Education	Spain	To know what aggressiveness is and to learn how to manage and cope with it through the hidden curriculum and emotions.	Analysis: - Observation Startup: - Workshops - Construction of standards - Dissemination	*●●●*
Oruche U. M. et al. (2013) [30]	Primary and Secondary Education	United States	To examine changes in personal strengths and family environment as predictors of behavioral and social functioning with adolescents who exhibit disruptive behavior.	- Questionnaires	*●●●*
Jones D. J. et al. (2014) [31]	Infant and Primary Education	United States	To implement a pilot study of a technology-based family training program to help children with disruptive behavior in order to increase the commitment of low-income families and thereby improve children’s behavior.	- Mobile phone	*●●●*
Montañez Gómez M. I. (2014) [32]	Primary Education	Spain	To develop and improve emotional intelligence competencies/skills and to offer home education guidelines.	- Questionnaires - Journal of sessions - Daily record sheet for the for daily life activities - Child Behavior Record Card	*●●○*
Yubero Jimenez S. et al. (2014) [33]	Primary and Secondary Education	Undetermined	To introduce children and adolescents to the analysis of peer relations and coexistence conflicts in educational centers through the reading of albums.	- Album	*●○○*
Burke J. D. et al. (2015) [34]	Primary Education	Canada	To reduce antisocial behavior in children aged 6-11.	- Questionnaires - Observation	*●●●*
Fernández Durán M. A. (2015) [35]	Secondary Education	Spain	To increase the social competence of secondary school students in order to promote their personal and social development.	- Communication, emotional control, problem-solving, and social skills techniques - Questionnaires	*●●●*
Mendoza González B. et al. (2015) [36]	Primary Education	Mexico	To examine the effectiveness of an intervention program based on the principles of Applied Behavioral Analysis to reduce children’s bullying behaviors in the school environment.	- Teacher training - Classroom rules - Social skills techniques - Motivation techniques -Emotional control techniques -Problem-solving skills techniques	*●●●*
Sánchez-Rivas E. et al. (2015) [37]	Primary Education	Spain	To contribute to the improvement of sport learning processes in the school environment.	- Questionnaires - Interviews - Observation - Follow-up: Checklists and logbooks for observation, disruptive behavior observation sheets, ludogram	*●●●*
Cherrés Sánchez M. (2018) [38]	Primary Education	Peru	To design a management and strengthening program to improve conflict resolution among students in the fifth grade of primary education.	- Bibliographic and newspaper sheets - Questionnaires	*●○○*
Hernández Ávila M. et al. (2016) [39]	Primary Education	Spain	To analyze teachers’ and students’ behavior in order to ascertain the degree of assimilation of the participatory or community model.	- Observation - “The traffic light”	*●●●*
Quintas Cid M. C. (2016) [40]	Secondary Education	Austria	To change the situation of maladaptation or social exclusion of adolescents.	- Systemic therapy - Experiential pedagogy - Gestalt psychology	*●●●*
Miranda Seguel C. F. et al. (2017) [41]	Secondary Education	Chile	To identify factors related to the interruption and maintenance of criminal behavior in adolescents.	- Statistical monitoring - Interviews (in-depth, with family models and the intervention team)	*●●○*
Aspiranti K. B. et al. (2018) [42]	Primary Education	Undetermined	To decrease inappropriate behaviors in primary education.	- “The color wheel”	*●●●*
Benavides Nieto A. (2018) [43]	Infant Education	Spain	To increase children’s social competence in order to prevent potential behavioral problems.	- Questionnaires - Semi-structured interviews	*●●●*
Eissa Saad M. A. (2018) [44]	Infant Education	United States	To examine the effectiveness of a program based on social skills training for the improvement of disruptive behaviors of children in early education.	- Instructions - Modeling - Social skills techniques (Role-play) - Feedback - Questionnaires	*●●●*
Mouton B. et al. (2018) [45]	Infant Education	Undetermined	To analyze the effect of family self-efficacy thinking changes on the behavior of children in early childhood education.	- Observation - Questionnaires	*●●●*
Obaco Puchaicela B. A. (2018). [46]	Secondary Education	Ecuador	To reduce bullying through a group awareness program.	- Questionnaires - Observation - Workshops	*●●●*
Puebla Juárez G. (2018) [47]	Primary Education	Spain	To identify, describe, and record a particular student’s disruptive behaviors in physical education classes, language, mathematics, and recess time, and to present a proposal for an intervention that aims to reduce the frequency of occurrence of these unwanted behaviors within the classroom, particularly in the subject of physical education.	Research: - Observation (systemic and non-systemic) - Questionnaires Proposal for intervention: - Factsheets - Creation of standards - Self-observation technique - Positive reinforcement	*●○○*
Roca Pacheco R. M. (2018) [5]	Primary Education	Colombia	To identify and describe the problems affecting Primary School students.	- Surveys (structured with closed questions)	*●●●*
Wora A. H. et al. (2018) [48]	Primary Education	United States	To examine the feasibility of implementing a pilot program based on culturally congruent mentoring, which aims to reduce the behaviors of elementary school students who exhibit repeated disruptive behavior.	- Observation - Questionnaires	*●●●*
Dadds M. R. et al. (2019) [49]	All stages	Australia	To examine the risk factors for abandonment of free, evidence-based, self-directed parental programs.	- Questionnaires	*●●●*
Díaz-López A. et al. (2019) [50]	Secondary Education	Spain	To evaluate the effect of a program based on emotional intelligence, on certain elements of school coexistence, bullying, as well as on motivation, empathy, and self-concept.	- Questionnaires	*●●●*
Fuster-Guilló A. et al. (2019) [51]	University Education	Spain	To examine the benefits generated by the teaching-learning process through gamification in computer engineering degrees, and to analyze the impact on motivation and satisfaction, as well as on academic performance.	- Questionnaires -Surveys	*●●●*
Goertz-Dorten A. et al. (2019) [52]	Primary Education	Germany	To decrease disruptive behaviors and behavioral problems in primary school students.	- Videos and cartoons - Social skills techniques (Role-play) - Questionnaires	*●●○*
González J. et al. (2019) [53]	Secondary Education	Spain	To analyze causal relationships between social interaction actions, the development of personality aspects, and the use of emotional intelligence skills in adolescents.	- Questionnaires	*●●●*
Ristkari T. et al. (2019) [54]	Infant Education	Finland	To describe how the Public Nursing System uses and gains experience from a working model that combines a psychosocial tool to identify disruptive behavior in four-year-olds and a family training program with internet support through telephone coaching.	- Electronic questionnaire	*●●●*
Romero Reignier V. et al. (2019) [55]	Primary Education	Undetermined	To present a proposal for intervention aimed at empowering Primary Education teachers to promote the emotional intelligence of their 5^th^ and 6^th^-grade students in order to reduce bullying among young people and improving school coexistence in general.	- Questionnaires	*○○○*

* Results related to the main objective: ●●●: Expected; ●●*○*: Partially positive; ●*○○*: No desirable; *○○○*: No result. It’s a new table reusing only some data in any publications.
